# The Expressions of TSLP, IL-33, and IL-17A in Monocyte Derived Dendritic Cells from Asthma and COPD Patients are Related to Epithelial–Macrophage Interactions

**DOI:** 10.3390/cells9091944

**Published:** 2020-08-22

**Authors:** Magdalena Paplinska-Goryca, Paulina Misiukiewicz-Stepien, Malgorzata Proboszcz, Patrycja Nejman-Gryz, Katarzyna Gorska, Rafal Krenke

**Affiliations:** 1 Department of Internal Medicine, Pulmonary Diseases and Allergy, Medical University of Warsaw, 02-091 Warsaw, Poland; malgorzata.proboszcz@wum.edu.pl (M.P.); patrycja.nejman-gryz@wum.edu.pl (P.N.-G.); katarzyna.gorska@wum.edu.pl (K.G.); rafal.krenke@wum.edu.pl (R.K.); 2 Postgraduate School of Molecular Medicine, Medical University of Warsaw, 02-091 Warsaw, Poland; paulina.misiukiewicz-stepien@wum.edu.pl

**Keywords:** asthma, COPD, dendritic cells, epithelium, TSLP, IL-33, IL-17A

## Abstract

Background. The cross-talk between the external and internal environment in the respiratory tract involves macrophage/dendritic cell (DC) transepithelial network. Epithelium triggers dendritic cell-mediated inflammation by producing thymic stromal lymphopoietin (TSLP), IL-33, and IL-17A. The study aimed to evaluate the expression of TSLP, IL-33, and IL-17A in human monocyte derived dendritic cells (moDCs) co-cultured with respiratory epithelium and monocyte derived macrophages (moMφs) in asthma, chronic obstructive pulmonary disease (COPD) and healthy controls. Methods. The study used a triple-cell co-culture model, utilizing nasal epithelial cells, along with moMφs and moDCs. Cells were cultured in mono-, di-, and triple-co-cultures for 24 h. Results. Co-culture with epithelium and moMφs significantly increased TSLP in asthma and did not change IL-33 and IL-17A mRNA expression in moDCs. moDCs from asthmatics were characterized by the highest TSLP mRNA expression and the richest population of TSLPR, ST2, and IL17RA expressed cells. A high number of positive correlations between the assessed cytokines and CHI3L1, IL-12p40, IL-1β, IL-6, IL-8, TNF in moDCs was observed in asthma and COPD. Conclusion. TSLP, IL-33, and IL-17A expression in moDCs are differently regulated by epithelium in asthma, COPD, and healthy subjects. These complex cell–cell interactions may impact airway inflammation and be an important factor in the pathobiology of asthma and COPD.

## 1. Introduction

Dendritic cells (DCs) are abundantly represented at the mucosal surfaces which are an interface between the external and internal environment. Having constant contact with antigens, DCs are thought to be one of the key regulators of the immune response initiation and modulation of inflammation in asthma and chronic obstructive pulmonary disease (COPD) [1,2]. Together with highly phagocytic macrophages, DCs interact closely with airway epithelium which forms the first line of contact barrier in the respiratory tract against inhaled pathogens, toxic particles, and allergens by expressing toll-like receptors (TLRs), C-type lectin, and protease-activated receptors [3]. Airway epithelium is able to produce and secrete antimicrobial and antiviral substances such as peptides or cytokines which can act against pathogens by themselves or could initiate cascade-based immune reactions [4]. As a result of allergen inhalation airway epithelial cells attract and activate DCs by producing pro-T helper 2 (Th2) polarizing cytokines and chemokines. DC activation can be mediated by uric acid production by epithelial cells which could be released in response to inhaled house dust mites and further stimulate cytokine production [5]. Epithelium derived cytokines (e.g., thymic stromal lymphopoietin (TSLP), IL-33, and IL-25) contribute to the immune response through creation of a positive feedback loop between airway epithelium and inflammatory cells [6,7]. Airway epithelium regulates the expression of crucial modulators of DC immunoresponse, like IL-1β, IL-6, IL-8, tumor necrosis factor alpha (TNF-α), IL-12p40, chitinase-3-like protein 1 (CHI3L1) [8,9,10,11,12]. The pathways of the epithelium–DC interplay seem to be crucial for understanding the process of allergic sensitization, including the role of genetic and environmental factors. 

TSLP and IL-33 are important modulators of DC function. TSLP-activated DCs induce CD4^+^ T cell proliferation and Th2 cell differentiation [13,14]. TSLP stimulates DCs to express the OX40 ligand (OX40L) which promotes TNF-α production in both Th1 and Th2 cell response [15]. Importantly, DCs not only respond to TSLP and IL-33 from external sources but can also produce these cytokines. TSLP shows an autocrine effect in DCs upregulating IL-1β, and hence IL-6 and IL-23 expression [16]. Mouse studies showed that DC-derived TSLP constrains Th17 cell development [17]. IL-33 is an important mediator of allergic response which activates DCs during antigen presentation and enhances their capacity for Th2 lymphocyte priming [18]. Importantly, IL-33 activates DCs by reprogramming naive T cells response that include IL-13 and IL-5 but not IL-4 production [19]. ST2/IL-33 signaling in DCs also increases IL-4, IL-5, IL-6, IL-13, CCL17, TNF-α, and IL-1β production [20]. In contrast to TSLP and IL-33, IL-17A is not an epithelial-derived cytokine but is an important activator of DC function associated with non-Th2 response. DCs are crucial for induction of Th17 subset production from T naïve lymphocytes. IL-17A modulates monocytes derived DC lipid metabolism and generates the foamy DCs phenotype (lipid-rich DCs) [21]. 

Considering the profound role of TLSP, IL-33, and IL-17A signaling in obstructive lung disease pathobiology, we sought to investigate whether the interactions between epithelium and macrophages impact TSLP, IL-33, and IL-17A expression in DCs in asthma and COPD. The aim of the study was to evaluate the impact of interactions between DCs and nasal epithelial cells co-cultured with macrophages on the expression of TSLP, IL-33, and IL-17A in asthma, COPD, and controls. 

## 2. Material and Methods

### 2.1. Overall Study Design

This was an experimental study that used in vitro cultures of monocyte derived dendritic cells (moDCs), monocyte derived macrophages (moMφs), and airway epithelial cells collected from asthmatics, COPD patients, and healthy subjects. The study included nasal brushing and peripheral blood collection from each patient. All procedures performed in this study were in accordance with the ethical standards of the institutional and/or national research committee and with the 1964 Helsinki declaration and its later amendments or comparable ethical standards. The study protocol was approved by the Ethics Committee of the Medical University of Warsaw (KB/120/2017) and informed written consent was obtained from all the participants. 

### 2.2. Patient Characteristics

The study involved 11 asthma patients, 11 patients with COPD, and 10 controls. Asthma and COPD were diagnosed and classified as recommended by current GINA and GOLD reports, respectively [22,23]. The major inclusion criteria were (1) diagnosis of asthma or COPD and (2) a stable course of the disease with no exacerbations in at least 4 weeks preceding the study onset. The major exclusion criteria included (1) severe asthma or severe COPD, (2) unstable or uncontrolled course of the disease, (3) treatment with systemic or nasal corticosteroids within 4 weeks before enrolment to the study. Atopic status was assessed by skin prick testing with a panel of 15 aeroallergens. The control group consisted of healthy smoking and non-smoking volunteers, with normal spirometry. The clinical characteristics of patients and controls are summarized in Table 1.

### 2.3. Monocyte Derived Dendritic Cell (moDC) and Monocyte Derived Macrophages (moMφ) Culture

Peripheral blood mononuclear cells (PBMCs) were isolated from freshly drawn venous blood by Lymphoprep (StemCell, Vancouver, British Columbia, Canada) centrifugation. The PBMC monocytes were achieved by adherence in Monocyte Attachment Medium (Promocell, Heidelberg, Germany) for 2 h. Monocyte derived dendritic cells (moDCs) were cultured in X-Vivo 20 medium (Lonza, Basel, Switzerland) supplemented with 40 ng/mL granulocyte-macrophage colony-stimulating factor (GM-CSF) (StemCell) for 8 days and 20 ng/mL IL-4 (PromoKine, Heidelberg, Germany) for 5 days and 50 ng/mL IL-1β (StemCell) and 50 ng/mL TNF-α (Biotechne, R&D Systems, Minneapolis, MN, USA) in the 6th day of culture. Monocyte derived macrophages (moMφs) were specialized from PBMC monocytes by stimulation with 20 ng/mL macrophage colony-stimulating factor (M-CSF) (StemCell) for 10 days in Macrophage Base Medium DXF (PromoCell). 

### 2.4. The Culture of Epithelial Cells in Air Liquid Interface (ALI) and Triple Co-Culture

Epithelial cells were obtained by nasal brushing (Cytobrush Plus GT, CooperSurgical, Trumbull, CT, USA). The culture of epithelial cells in ALI condition as well as triple co-cultures were prepared as previously described [24]. Each triple-cell co-culture model contained three different cell types obtained from the same patient. ALI epithelial cells were cultured with or without stimulation with 50 ng/mL of IL-13 (PromoKine) or 10 µg/mL poly I:C (Sigma Aldrich, St. Louis, MO, USA) for 24 h in a scheme as follows: (1) epithelial cells, (2) epithelial cells+moDCs (co-culture), (3) epithelial cells+ moMφs (co-culture), (4) moDCs+epithelial cells+ moMφs (triple co-culture). 

### 2.5. RNA Isolation, cDNA Synthesis, and Real Time PCR

Total RNA was isolated from the DCs using Trizol (Sigma Aldrich). Ten microliters of total RNA was used for reverse transcription (Thermo Fisher Scientific, Waltham, MA, USA). Real-time PCR measurements were performed with an ABI-Prism 7500 Sequence Detector System (Thermo Fisher Scientific, Applied Biosystems, Waltham, MA, USA). For PCR reaction 0.8 µL of cDNA was amplified in 16 µL PCR final volume, containing a TaqMan master mix (Thermo Fisher Scientific) with 150 nM of specific primers and 100 nM of probe (TSLP Hs00263639_m1, IL-33 Hs00369211_m1, IL-6 Hs00174131_m1, IL-1β Hs01555410_m1, TNF-α Hs00174128_m1, CHI3L1 Hs01072228_m1, IL12p40 F:CGGTCATCTGCCGCAAA, R: TGCCCATTCGCTCCAAGA, P: 6-FAM-CGGGCCCAGGACCGCTACTATAGCT-TAMRA, IL-8 F: AGCACACAAGCTTCTAGGACAAGA R: GGAAGTCATGTTTACACACAGTGAGA, P:6-FAM CCAGGAAGAAACCACCGGAAGGAACC-TAMRA, IL17A F: AGGAATCACAATCCCACGAAAT, R:GGTGAGGTGGATCGGTTGTAGT, P: 6-FAM AGGACAAGAACTTCCCCCGGACTGTG-TAMRA, 18S rRNA Hs99999901_s1, Thermo Fisher Scientific). Each sample was measured in duplicate. The results were expressed as relative quantification units (fold change). Relative quantification values were calculated by the 2^-∆∆CT^ method. 18S rRNA was applied for each sample as an internal control in order to normalize gene expression levels. moDCs without co-cultivation were used as a calibrator.

### 2.6. Flow Cytometry Analysis

Human TruStain FcX (Biolegend, San Diego, CA, USA) (3 μL per 100 μL of sample) was added to moDCs to block non-specific bindings. Cells were stained with antibodies against the surface binding molecules: TSLPR BV421 (clone 1F11/TSLPR), CD45 APC-H7 (clone 2D1), CD14 BV605 (clone M5E2), CD83 BV510 (clone HB15e), CD11B APC-R700 (Clone M1/70) (BD Biosciences, San Jose, CA, USA) ST2 APC (Biotechne, R&D Systems, Minneapolis, MN, USA), IL17RA PE (clone REA290) (Miltenyi Biotec, Bergisch Gladbach, Germany) in BD Horizon Brilliant Stain Buffer (BD Biosciences) and incubated for 20 min in the dark at room temperature. 

Cells were analyzed by flow cytometry using a FACS Celesta instrument (BD Biosciences) equipped with blue (488-nm), violet (405-nm), and red (640-nm) lasers. Unstained cells and compensation beads (BD Biosciences) were used to set voltages and create single stain negative and positive controls. Compensation was set to account for spectral overlap between the seven fluorescent channels used in the study. Samples were examined by side scatter area (SSC-A) versus forward scatter area (FSC-A), then using forward scatter height (FSC-H) versus FSC-A to select single cells, eliminating debris and clumped cells from the analysis. At least 50,000 cells in the target gate were collected. The results are presented as percentage of positive cells of the respective gate. moDCs was characterized as CD45+CD14-CD11b+CD83+ cell population.

### 2.7. Statistical Analysis

Statistical analysis was performed with the use of Statistica 13.3 software package (StatSoft Inc., Tulsa, OK, USA). The Kruskal–Wallis test was used to assess differences between continuous variables in the three study groups. The Mann–Whitney U test was applied for pairwise comparisons. Pearson Chi-square test was used to compare inter-group differences between categorical variables. Correlations between variables were analyzed with Spearman’s rank test. Linear regression model was fitted with a given cytokine as dependent variable and diagnosis (control/asthma/COPD) and BMI as explanatory variables. Separate models were fitted for each cytokine and setting (moDCs, moDCs/epithelium, moDCs/epithelium+moMφs). Calculations were performed with R (version 3.6.1). Results are given as median and interquartile range (IQR). Differences were considered statistically significant at *p* < 0.05. 

## 3. Results

### 3.1. moDCs TSLP, IL-33, and IL-17A mRNA Expression in Multi Co-Cultures in Control Group

**Figure 1:**
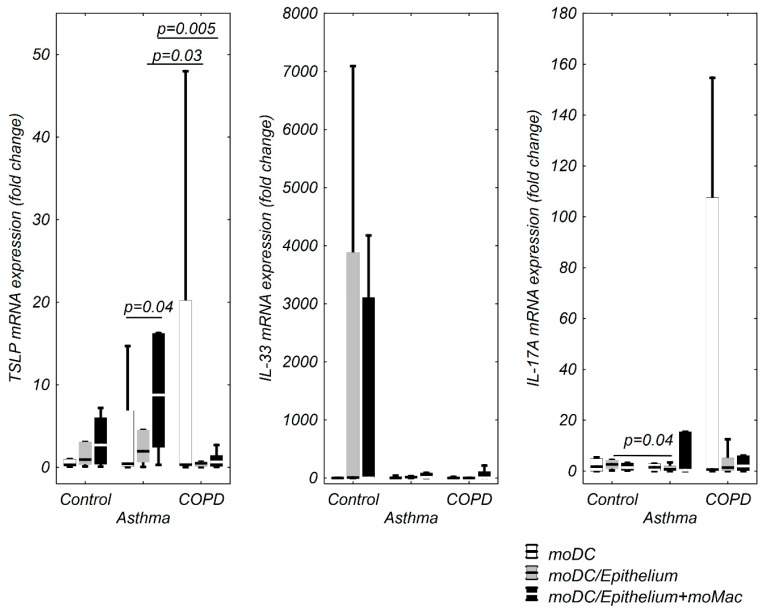
Thymic stromal lymphopoietin (TSLP), IL-33, and IL-17A mRNA expression in monocyte derived dendritic cells (moDCs) in multi co-culture schemes in control subjects, patients with asthma, and patients with chronic obstructive pulmonary disease (COPD). The data are shown as non-outlier range (whiskers), interquartile range (box), and median (line).

Co-cultivation of moDCs with epithelium (0.94 fold change (0.37–3.07 fold change)) and epithelium+moMφs (2.69 fold change (0.38–6.02 fold change)) insignificantly upregulated TSLP mRNA expression compared to moDCs alone (0.30 fold change (0.13–1.01 fold change)). The highest (without significance) TSLP mRNA expression was observed in moDCs co-cultivated with epithelium+moMφs (Figure 1). There were no significant IL-33 mRNA changes in the control group (Figure 1).

IL-17A mRNA expression was higher in moDCs/epithelium (2.73 fold change (1.73–4.36 fold change)) and lower in moDCs/epithelium+moMφs (0.95 fold change (0.27–3.28 fold change)) compared to moDCs alone (1.74 fold change (0.003–4.95 fold change)). However, the differences were insignificant (Figure 1). 

**Figure 2:**
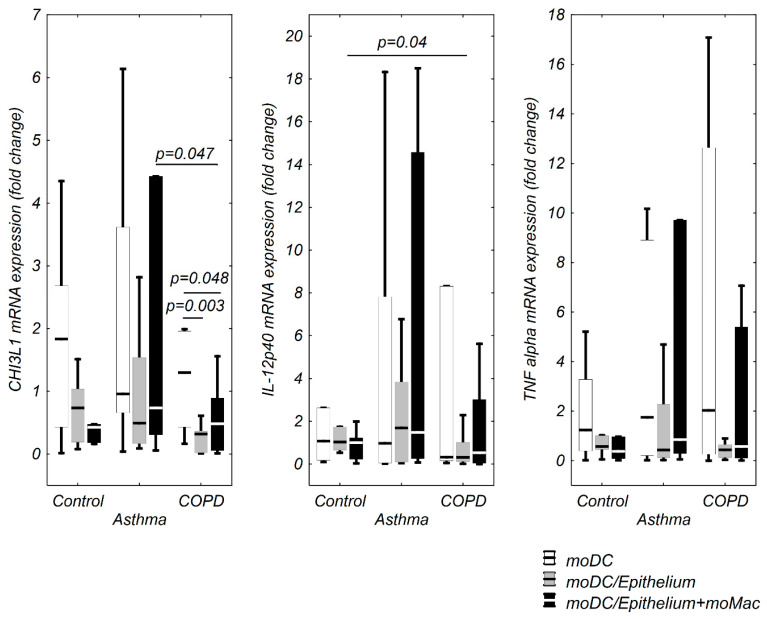
Chitinase-3-like protein 1 (CHI3L1), IL-12p40, and tumor necrosis factor alpha (TNF-α) mRNA expression in moDCs in multi co-culture schemes in control subjects, patients with asthma, and patients with COPD. The data are shown as non-outlier range (whiskers), interquartile range (box), and median (line).

**Figure 3:**
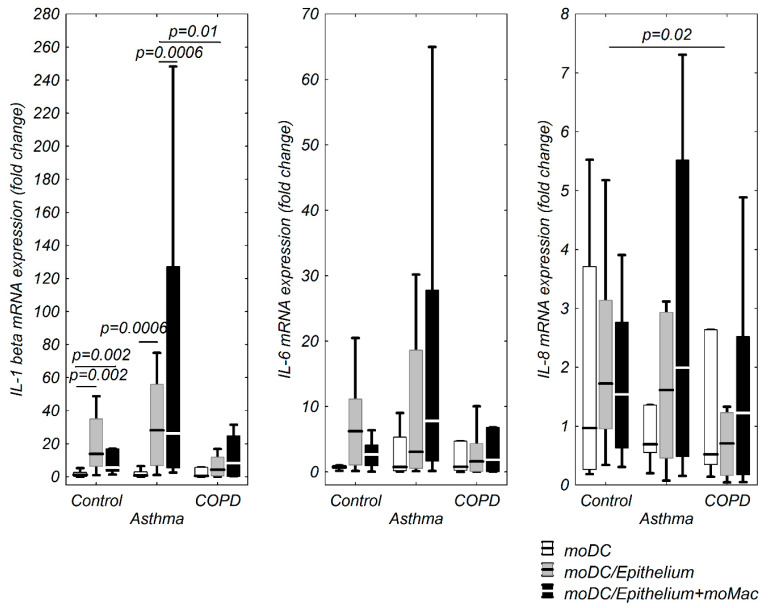
IL-1β, IL-6, and IL-8 mRNA expression in moDCs in multi co-culture schemes in control subjects, patients with asthma, and patients with COPD. The data are shown as non-outlier range (whiskers), interquartile range (box), and median (line).

CHI3L1, IL-12p40, IL-1β, IL-6, IL-8, TNF-α mRNA expression in moDCs in the evaluated co-cultures and stimulation model is shown in Figure 2 and Figure 3. Only a few significant correlations between TSLP, IL-33, and the investigated cytokines were observed in the control group. TSLP mRNA expression correlated positively with CHI3L1 and IL-1β in moDCs alone, negatively with IL-12p40 in moDCs co-cultivated with epithelium and strongly, positively with IL-33 mRNA expression in moDCs from triple co-cultures (Table 2).

### 3.2. moDCs TSLP, IL-33, and IL-17A mRNA Expression in Multi Co-Cultures in the Asthma Group

We found an elevated TSLP mRNA expression in moDCs co-cultivated with both epithelium (1.94 fold change (0.63–4.53 fold change)) and moMφs (8.76 fold change (2.42–16.27 fold change)) compared to moDCs alone (0.42 fold change (0.11–6.89 fold change)), however, only the difference between moDCs and moDCs/epithelium+moMφs was significant (Figure 1). The asthmatic moDCs were characterized by the highest TSLP mRNA expression in di- and triple-co culture compared to respective COPD groups (Figure 1).

An insignificant increase of IL-33 mRNA expression was noted in moDCs co-cultivated with epithelium (15.10 fold change (3.66–21.20 fold change)) as well as with epithelium+moMφs (11.32 fold change (0.24–89.83 fold change)) compared to moDCs alone (2.26 fold change (0.37–23.37 fold change)) (Figure 1). IL-17A mRNA expression was insignificantly decreased in all moDCs from co-culture schemes compared to moDCs alone. The lowest IL-17A mRNA expression in asthma was observed in moDCs cultured with epithelium compared to respective co-cultivation scheme in the control group (Figure 1).

TSLP mRNA expression significantly correlated with IL-12p40 mRNA expression in moDCs cultured alone. The co-cultivation of moDCs in triple-co-culture affected the interactions between cytokine mRNA expression and resulted in an increased number of significant (all positive) correlations, with the strongest correlation between TSLP and IL-6 mRNA (Table 3). There was a strong positive correlation between IL-17A mRNA expression and IL-12p40, IL-1β, IL-6 in moDCs alone and with IL-12p40, IL-6, and IL-33 in moDCs from triple co-cultures. Such a relationship was not observed in moDCs/epithelium group (Table 3).

### 3.3. TSLP, IL-33, and IL-17A mRNA Expression in moDCs in Multi Co-Culture Schemes in the COPD Group

There were no differences in TSLP mRNA expression in COPD group regardless of the co-culture type used. Likewise, IL-33 mRNA expression remained at the same level in all co-culture schemes (Figure 1). We did not notice any differences in IL-17A mRNA expression in moDCs (Figure 1).

Despite the lack of significant changes in TSLP, IL-33, and IL-17A mRNA expression in the evaluated co-cultures and stimulation models, the largest number of significant correlations between cytokine mRNA expressions was observed in the COPD group. TSLP mRNA expression correlated positively with most of the assessed cytokines in every co-culture scheme but noteworthy, a strong correlation with TNF-α mRNA expression occurred in all moDCs (with or without co-cultivation) (Table 4). Interestingly, IL-33 mRNA expression correlated positively with CHI3L1, IL-12p40, IL-1β, and TSLP only in moDCs cultured alone. This phenomenon was not observed in any other co-culture type (Table 4). IL-17A mRNA expression correlated positively with almost all cytokines (except for IL-33) in moDCs alone. In moDCs from co-cultures, the number of significant correlations was lower (Table 4).

The results regarding TSLP, IL-33, and IL-17A as well as CHI3L1, IL-12p40, TNF-α IL-1β, IL-6, and IL-8 mRNA expression in moDCs after IL-13 (of pro-allergic) or polyinosinic:polycytidylic acid (poly I:C) (viral like) stimulation in di- and triple-co-culture schemes in control subjects, patients with asthma, and patients with COPD are described in the Supplementary Materials and shown in Figures S1–S3.

### 3.4. The Impact of BMI on TSLP, IL-33, and IL-17A mRNA Expression in moDCs

Obesity did not influence TSLP and IL-33 mRNA expression in any of the evaluated groups, but BMI might impact the IL-17A mRNA expression in asthma. BMI correlated significantly with IL-17A mRNA expression in moDCs co-cultivated with epithelium (*r* = −0.745, *p* = 0.010) in the asthma group only. For this reason, we performed the linear regression with BMI and diagnosis as independent variable. We found that IL-17A mRNA expression was not decreased in the asthma group compared to controls in moDCs co-cultivated with epithelium (*p* = 0.285), but revealed a significantly lower IL-17A mRNA expression in moDCs alone from asthma (*p* = 0.005) and COPD (*p* = 0.049) patients compared to controls. IL-17A mRNA expression was significantly negatively associated with BMI (*p* = 0.00018) in this setting.

### 3.5. The Expression of TSLPR, ST2, and IL-17RA on moDCs in Various Co-Cultures from Control, Asthma, and COPD Groups

The proportion of TSLPR+, ST2+, and IL-17RA+ moDCs was similar in moDCs regardless of the co-culture model. Noteworthy, the number of TSLPR+, ST2+, and IL-17RA+ moDCs differed between asthma, COPD, and controls (Table 5). An increased proportion of all analyzed TSLPR+ moDCs was observed in asthma (*p =* 0.00002) and COPD compared (*p =* 0.002) to control with the highest number of moDCs expressed TSLPR in the asthma group. In a more detailed analysis, we found an elevated number of TSLPR+ cells in moDCs without co-cultivation in asthma (*p* = 0.02) and COPD (*p* = 0.009) compared to controls. In moDCs from triple-co-culture a higher number of TSLPR+ cells was found in asthma compared to the control group (*p =* 0.03). Similarly, the asthma group was characterized by the largest number of ST2+ moDCs significant in the group of all analyzed cells (*p =* 0.04 compared to controls). The increased proportion of all IL17RA+ moDCs was found in asthmatics (*p* = 0.002) and COPD patients (*p* = 0.04) compared to controls (Table 5).

## 4. Discussion

To our knowledge, this is the first study that evaluated the impact of interactions between moDCs and epithelial cells co-cultured with moMφs on TSLP, IL-33, and IL-17A expression in asthma and COPD. We found that co-culture with epithelium and moMφs significantly increased TSLP mRNA in the asthma group and to a lesser extent or did not change IL-33 and IL-17A mRNA expression in moDCs. We also demonstrated substantial differences in mRNA expression of the evaluated cytokines between obstructive lung diseases and controls as well as between asthma and COPD. moDCs from asthmatics were characterized by the highest TSLP mRNA expression and the largest number of TSLPR+, ST2+, and IL17RA+ cells. Although we did not observe any changes in TSLP, IL-33, or IL-17A mRNA expression in moDCs from COPD patients in any of the used co-culture or stimulation model, an interesting finding is the high number of positive correlations between the assessed cytokines and markers of immunoresponse in moDCs from triple co-cultures. Further, the study showed an elevated number of TSLPR and IL-17RA expressed moDCs from COPD patients suggesting an important (but different than in asthma) immunological reaction of DCs in COPD. Taken together, the results of our work suggest that different regulation of DC function in obstructive lung diseases may be related to complex cell–cell interactions with the special regard to airway epithelium dysfunction creating the pattern of biological reactions.

The cross-talk between DCs and airway epithelial cells is controlled by cytokines released from airway epithelial cells. TSLP expression is increased in asthmatic airways and correlates with disease severity [25]. The impact of epithelial derived TSLP on DCs has been well established [26]. TSLP activated DC primed naïve T-lymphocytes to produce Th2 cytokines (IL-4, IL-5, IL-13) and TNF-α but downregulated IL-10 and interferon gamma (IFN-γ) [27]. Our previous study showed that TSLP expression in ALI-cultured epithelial cells was related to epithelial–DC–macrophage interaction and indicated an impaired function of asthmatic epithelial cells [24]. On the other hand, the role of DC-derived TSLP remains unclear. It is speculated that TSLPR signaling on DCs might regulate Th1- and Th17-cell differentiation. Mouse model studies showed that TSLP produced by gut DCs acts directly on T cells by reducing their capacity to produce IL-17 and acting as an anti-inflammatory agent [17]. The results of our study clearly demonstrated that moDCs from asthma are the richest source of TSLP, especially in triple co-culture and are the most potent responders for TSLP action. The results of our study indicated a low expression of TSLP in moDCs from COPD patients. An elevated amount of TSLP protein was found in COPD nasal epithelial cells [24]. Some authors suggest that oxidative stress which is present locally in the upper and lower respiratory epithelium of tobacco smokers may augment TSLP mRNA expression and promote the development of Th-2 dependent inflammatory response in COPD [28]. We suggest that since the moDCs used in our study were not stimulated with cigarette smoke extract, we did not find an increased expression of TSLP in moDCs from COPD patients. The result of our study confirmed that epithelial/moDCs co-cultivation significantly changed the expression of many mediators in DCs and that cell–cell cross-talk dynamically directs the immunological reaction [29]. Interestingly, TSLP expression in moDCs from triple co-culture correlated positively with IL12p40, IL-6, and TNF-α in asthma, whereas in COPD with CHI3L1, IL12p40, IL-6, IL-8, TNF-α, and IL-1β suggesting a different pathway of immune response in asthma and COPD. Our finding of the positive correlation between mRNA expression of TSLP and IL-1β in moDCs alone of control and COPD subjects, as well as even a stronger relationship in moDCs after co-cultivation with epithelium and moMφs in COPD seems to confirm the results of Elder at al. who highlighted the link between TSLP and IL-1β [11,16]. These results may indicate the reprogrammed function of DCs associated with Th1 type of immunological reaction and probably with the response to bacterial/fungal colonization of the airways frequently observed in COPD. 

The role of DCs in COPD is the subject of numerous investigations. The altered role of DCs in COPD patients may be related to cigarette smoke and oxidative stress but also to disease progression per se. Cigarette smoke activates DCs, changes their migration capacity, and impacts maturation of DCs [30,31]. DCs produce a high amount of pro-fibrotic agents and impact local inflammation by secretion of various cytokines. These immunological actions of DCs may be related to the pathology of emphysema [32]. As COPD progresses, the more mature activated DCs attract airways leading to destructive immune response. Freeman et al. showed that an increased number of CD83 and CD80 pulmonary DCs correlated with worsening COPD severity and positively correlated with expression of CD69 on lung CD4 T-cells which indicated the DCs–T lymphocyte active interaction [33]. Many associations between TSLP and COPD are still unexplained. Elevated levels of mRNA and protein of TSLP were found in COPD patients compared to healthy non-smokers [34]. A variable TSLP gene activation in the presence of heavy smoking may contribute to this progression into specific COPD phenotypes (eosinophilic) [35]. The possible explanation of increased TSLP expression in the airways of COPD could be related to the fact that IL-1β and TNF-α induce TSLP expression via (p38, p42/p44) MAPK signaling pathway [36]. Our study demonstrated that the main producers of airway TSLP are epithelial cells [24] whereas in contrast with asthma DCs, even after co-stimulation with other cells, COPD DCs produce a very low amount of this cytokine. However, the variable changes in expression of other DCs immunomodulators after epithelium and moMφs co-cultivation reflect the complex pattern of DC biology in COPD airways.

Limited information is available on the role of IL-33 in DC function. IL-33 acts in an ST2-dependent manner, as a maturation factor for DCs via the upregulation of CD80, CD40, and OX40L and by triggering GM-CSF production by other bone marrow leukocyte populations [37,38]. The results of mouse studies showed that IL-33 expression in DCs is induced in response to bacterial or allergic stimuli. DCs produce IL-33 via TLR/NF-κB signaling pathway after lipopolysaccharide (LPS) and flagellin stimulation [20]. Another study demonstrated that DCs are able to produce IL-33 after IgG immune complex and house dust mite stimulation [39]. Our study showed that IL-33 is expressed in DCs only after co-cultivation with epithelial cells+/-moMφ or by IL-13 or poly I:C stimulation, but the difference in relation to moDCs alone was insignificant. An interesting observation is the correlation between IL-33 and CHI3L1 (YKL-40) mRNA expression in asthma and COPD suggesting possible common pathways of these two mediators. The largest number of ST2 expressed moDCs in asthma may suggest the important effect of epithelial derived IL-33 on DCs response in this disease.

Our study revealed a possible link between DCs and the IL-17A pathway. The common role of DCs and IL-17A is associated with Th17 cell differentiation. Th17 differentiation is promoted by IL-6 and TGF-β together with IL-23 and IL-1β which support the full maturation of Th17 cells. All these cytokines can be released by DCs. Classically IL-17A secretion is IL-23-dependent, however, an IL-23-independent production of IL-17A may occur in response to presentation of glycolipid antigens by CD1d [40]. It is speculated that Th17 cells are not the only source of IL-17A but other cells of the innate immune system also produce considerable amounts of this cytokine [41]. Here, for the first time, we showed that moDCs after epithelial or epithelial/moMφ co-cultivation are able to express IL-17A mRNA. Interestingly, the expression of IL-17A in moDCs was lower in asthma and COPD compared to controls. This effect was associated with BMI in asthma and COPD patients. The most prominent function of IL-17 is the induction of antimicrobial responses against extracellular pathogens which include recruitment of neutrophils to the site of infection [42]. Additionally, IL-17A may affect epithelial cells to promote tissue remodeling [43]. The results of our study suggest the possible dynamic interactions between immunological and structural cells of the respiratory tract that create the response pattern for pathogen infection: epithelial cells stimulated with bacterial load signal DCs which may produce IL-17A for neutrophil attraction necessary for clearing microorganisms. Importantly, the largest number of IL-17RA+moDCs was observed in asthma and COPD, suggesting the altered function of DC-IL-17A signaling in these diseases. It is worth noticing that IL-17RA together with IL-17RB is an important part of IL-25 signaling—an epithelial derived alarmin significant in asthma pathobiology. Taken together our findings regarding IL-17A-moDC common pathway needs further investigations.

Although our study provided some new information about TSLP, IL-33, and IL-17A expression in moDCs, several limitations must be addressed before more definitive conclusions will be drawn. The major one is the in vitro character of the study which used DCs differentiated from peripheral blood monocytes. These cells mimic DCs (functionally and phenotypically) but still, they are not pulmonary resident DCs [44]. Human DCs are difficult to study in vivo. Some authors used ex vivo studies which require an invasive method of lung tissue obtaining and enzymatic or non-enzymatic isolation from lung tissue specimens. It is worth noticing that the phenotype of tissue isolated DCs is very sensitive and can rapidly change upon the absence of biological background. Therefore, we adapted one-to-three cell type co-culture model of the airways using non-invasively obtained blood and nasal samples. We believe this model reflects only some cell-to-cell interactions critical for airway cellular signaling. We are aware that moDCs in our co-culture scheme were incubated in medium dedicated for epithelial cells, what may have impacted the moDC polarization pattern. In study of Blom et al. who used a similar triple-co-culture system but with a monolayer epithelial culture (not ALI cultured) the epithelial medium (MEM) was added to the bottom chamber where moDCs were attached [45]. This methodology bias may influence the results of the current study, however, the obtained results revealed the basal pathways in moDCs which are impacted by epithelial secretome of controls, patients with asthma, and patients with COPD. It should also be borne in mind that data concerning the subject of this study is scarce. Hence, the new information we provide may help with better exploration of the immunological structure of the cell–cell interaction pattern.

## 5. Conclusions

DCs may be a potential non-epithelial source of TSLP or IL-33 in the airways. Co-cultivation of moDCs with epithelial cells and moMφs increased TSLP mRNA expression with the most pronounced effect observed in asthma. Asthma and COPD DCs differ in the pattern of immunological response and DC interactions with structural cells. Interestingly, we found that after contact with epithelial cells moDCs are able to express IL-17A mRNA. Our results suggest a possible dynamic cell–cell cross-communication which creates an immunological response in the airways. We believe our study may help to understand the role of TSLP, IL-33, and IL-17A in a positive feedback loop between the airway epithelium and inflammatory cells infiltrating the airways in obstructive lung diseases.

## Figures and Tables

**Table 1:** Major characteristics of three groups included in the study, i.e., healthy subjects, asthma, and chronic obstructive pulmonary disease (COPD) patients.

	Control *n* = 10	Asthma *n* = 11	COPD *n* = 11	Overall *p*-Value ^&^	Pairwise *p*-Value *		
					Asthma vs. Control	COPD vs. Control	Asthma vs. COPD
Age (years)	30 (25–40)	55 (43–71)	65 (60–74.4)	0.001	0.0004	0.0001	0.084
Gender (F/M)	8/2	6/5	7/4	0.450			
BMI (kg/m^2^)	21.7 (19.8–23.6)	27.5 (26.9–29.8)	28.3 (24.8–29.4)	0.024	0.029	0.010	0.898
Atopy (n)	2	7	3	0.103			
Smoking exposure (pack-years)	0 (0–1)	0 (0–0)	47.4 (30–52.5)	<0.0001	0.436	<0.0001	0.0008
FEV_1_ (% predicted)	107.5 (104–110)	79 (75–86)	58 (48–72)	<0.0001	<0.0001	0.0002	0.0066
FEV_1_/VC (%)	94.0 (86.0–107)	81.5 (76–91)	53 (50–64)	<0.0001	0.023	0.0001	0.0002
FeNO (ppb)	15.0 (12.6–16.3)	28.0 (13.5–59.9)	18.0 (14.6–23.1)	0.076	0.055	0.121	0.237
ACT (points)	N.A.	22 (19–24)	N.A.	N.A	N.A.	N.A.	N.A.
ICS treatment (n)	N.A.	6	0	N.A	N.A.	N.A.	N.A.
LABA/LAMA treatment (n)	N.A.	9	10	N.A	N.A	N.A	N.A
CAT (points)	N.A.	N.A.	10 (8–12)	N.A	N.A.	N.A.	N.A.
mMRC (points)	N.A.	N.A.	2 (1–2.5)	N.A	N.A.	N.A.	N.A.

Data are presented as median (IQR) or n. ACT—asthma control test, BMI—body mass index, FeNO—fractional exhaled nitric oxide, FEV_1_—forced expiratory volume at first second, ICS—inhaled corticosteroids, LABA—long-acting beta agonists, LAMA—long-acting muscarinic antagonists, mMRC—modified Medical Research Council, N.A—not applicable, VC—vital capacity. ^&^ Kruskal Wallis or Chi square test, * Mann–Whitney U test.

**Table 2:** Correlations between thymic stromal lymphopoietin (TSLP), IL-33, IL-17A, and chitinase-3-like protein 1 (CHI3L1), IL-12p40, IL-1β, IL-6, IL-8, tumor necrosis factor alpha (TNF-α) mRNA expression in monocyte derived dendritic cells (moDCs) multi co-cultures of control subjects.

Control	moDCs						moDCs/Epithelium						moDCs/Epithelium+moMφs					
	TSLP		IL-33		IL-17A		TSLP		IL-33		IL-17A		TSLP		IL-33		IL-17A	
	R	*p*-Value	R	*p*-Value	R	*p*-Value	R	*p*-Value	R	*p*-Value	R	*p*-Value	R	*p*-Value	R	*p*-Value	R	*p*-Value
CHI3L1	0.83	0.01	0.17	0.67	0.25	0.49	−0.27	0.49	−0.50	0.21	−0.03	0.93	−0.10	0.82	−0.38	0.31	−0.14	0.76
IL-12p40	−0.18	0.64	−0.35	0.36	−0.53	0.12	−0.70	0.04	−0.69	0.06	−0.20	0.61	−0.69	0.06	−0.53	0.14	−0.14	0.76
IL-1β	0.72	0.03	-0.03	0.93	0.08	0.83	0.10	0.80	0.19	0.65	−0.03	0.93	0.31	0.46	0.10	0.80	−0.32	0.48
IL-6	−0.17	0.67	0.28	0.46	0.58	0.08	0.57	0.11	0.40	0.32	0.62	0.08	0.69	0.06	0.18	0.64	0.18	0.70
IL-8	0.28	0.46	−0.08	0.83	0.35	0.33	0.17	0.67	−0.24	0.57	0.30	0.43	0.31	0.46	0.15	0.70	0.00	1.00
TNF-α	0.17	0.67	0.03	0.93	−0.07	0.85	−0.47	0.21	−0.17	0.69	0.27	0.49	−0.48	0.23	−0.07	0.86	−0.75	0.05
TSLP			0.17	0.69	0.22	0.58			0.75	0.05	0.50	0.21			0.93	0.003	0.50	0.39
IL-33	0.17	0.69			0.52	0.15	0.75	0.05			0.04	0.94	0.93	0.003			−0.20	0.70
IL-17A	0.22	0.58	0.52	0.15			0.50	0.21	0.04	0.94			0.50	0.39	−0.20	0.70		

**Table 3:** Correlations between TSLP, IL-33, IL-17A and CHI3L1, IL-12p40, IL-1β, IL-6, IL-8, TNF-α mRNA expression in moDCs multi co-cultures of asthma patients.

Asthma	moDCs						moDCs/Epithelium						moDCs/Epithelium+moMφs					
	TSLP		IL-33		IL-17A		TSLP		IL-33		IL-17A		TSLP		IL-33		IL-17A	
	R	*p*-Value	R	*p*-Value	R	*p*-Value	R	*p*-Value	R	*p*-Value	R	*p*-Value	R	*p*-Value	R	*p*-Value	R	*p*-Value
CHI3L1	0.07	0.85	0.43	0.24	0.42	0.20	−0.52	0.13	0.22	0.58	−0.06	0.85	0.52	0.13	0.80	0.01	0.48	0.13
IL-12p40	0.65	0.04	0.33	0.38	0.72	0.01	0.48	0.16	−0.13	0.73	0.54	0.09	0.77	0.01	0.57	0.11	0.70	0.02
IL-1β	0.61	0.06	0.48	0.19	0.70	0.02	−0.39	0.26	0.00	1.00	0.50	0.12	0.41	0.24	0.62	0.08	0.52	0.10
IL-6	0.43	0.21	0.15	0.70	0.75	0.01	0.05	0.88	−0.27	0.49	0.54	0.09	0.82	0.00	0.52	0.15	0.67	0.02
IL-8	−0.12	0.75	0.55	0.12	0.40	0.22	−0.20	0.58	−0.03	0.93	0.50	0.12	0.42	0.23	0.72	0.03	0.57	0.07
TNF-α	0.35	0.36	0.55	0.16	0.22	0.53	−0.03	0.93	−0.48	0.23	0.48	0.16	0.72	0.03	0.67	0.07	0.31	0.38
TSLP			0.31	0.46	0.38	0.28			−0.13	0.73	0.32	0.37			0.43	0.29	0.25	0.49
IL-33	0.31	0.46			0.37	0.33	−0.13	0.73			−0.23	0.55	0.43	0.29			0.68	0.04
IL-17A	0.38	0.28	0.37	0.33			0.32	0.37	−0.23	0.55			0.25	0.49	0.68	0.04		

**Table 4:** Correlations between TSLP, IL-33, IL-17A and CHI3L1, IL-12p40, IL-1β, IL-6, IL-8, TNF-α mRNA expression in moDCs multi co-cultures of COPD patients.

COPD	moDCs						moDCs/Epithelium						moDCs/Epithelium+moMφs					
	TSLP		IL-33		IL-17A		TSLP		IL-33		IL-17A		TSLP		IL-33		IL-17A	
	R	*p*-Value	R	*p*-Value	R	*p*-Value	R	*p*-Value	R	*p*-Value	R	*p*-Value	R	*p*-Value	R	*p*-Value	R	*p*-Value
CHI3L1	0.53	0.10	0.62	0.04	0.63	0.04	0.67	0.03	0.10	0.82	0.61	0.05	0.70	0.03	0.55	0.16	0.43	0.19
IL-12p40	0.58	0.06	0.75	0.01	0.67	0.02	0.65	0.04	0.38	0.35	0.48	0.13	0.79	0.01	0.10	0.82	0.62	0.04
IL-1β	0.66	0.03	0.75	0.01	0.75	0.01	0.52	0.13	−0.10	0.82	0.68	0.02	0.87	0.001	0.19	0.65	0.54	0.09
IL-6	0.46	0.15	0.44	0.18	0.65	0.03	0.54	0.11	0.07	0.87	0.66	0.03	0.85	0.002	−0.33	0.42	0.71	0.01
IL-8	0.53	0.10	0.45	0.17	0.65	0.03	0.70	0.03	0.19	0.65	0.61	0.05	0.72	0.02	0.29	0.49	0.65	0.03
TNF-α	0.65	0.03	0.54	0.09	0.73	0.01	0.88	0.002	0.11	0.82	0.61	0.06	0.88	0.001	−0.33	0.42	0.51	0.11
TSLP			0.65	0.03	0.78	0.00			0.29	0.49	0.56	0.09			−0.14	0.74	0.50	0.14
IL-33	0.65	0.03			0.47	0.14	0.29	0.49			−0.45	0.26	−0.14	0.74			−0.12	0.78
IL-17A	0.78	0.00	0.47	0.14			0.56	0.09	−0.45	0.26			0.50	0.14	−0.12	0.78		

**Table 5:** TSLPR, ST2, IL17RA expression on moDCs upon co-cultivation with epithelium and monocyte derived macrophages (moMφs) in the multi-cell model culture of controls, patients with asthma and patients with COPD.

	Controls	Asthma	COPD	*p*-Value ◊
**TSLPR**				
moDC	10.45 (7.30–12.50) *#	28.20 (28.20–29.50) *	21.90 (18.10–22.10) #	0.009
moDC/epithelium	9.05 (4.80–13.50)	36.60 (11.70–46.50)	12.60 (12.20–17.70)	0.12
moDC/epithelium/moMφ	10.10 (6.20–16.70) *	28.20 (19.00–31.90) *	18.90 (12.70–33.00)	0.06
Summed cells	9.95 (6.20–13.50) *#	28.20 (16.00–9.30) *^	18.10 (12.60–23.50) #^	0.001
**ST2**				
moDC	24.05 (17.30–31.30)	27.60 (27.00–32.40)	26.20 (18.60–39.80)	0.51
moDC/epithelium	29.30 (18.10–33.80)	40.90 (31.50–43.80)	39.90 (20.10–43.40)	0.39
moDC/epithelium/moMφ	30.55 (19.60–32.90)	35.10 (29.70–35.80)	40.30 (17.80–44.20)	0.62
Summed cells	28.30 (18.10–33.60) *	32.40 (27.00–42.50) *	39.80 (17.80–44.20)	0.16
**IL17RA**				
moDC	39.50 (39.50–52.20)	64.80 (60.40–65.30)	58.60 (47.00–64.50)	0.11
moDC/Epithelium	37.65 (23.30–57.00)	60.80 (49.60–61.40)	54.60 (39.80–61.10)	0.30
moDC/Epithelium/moMφ	28.60 (23.30–45.10)	51.60 (47.40–52.70)	63.80 (26.80–64.80)	0.15
Summed cells	37.15 (23.30–52.20) *#	60.40 (47.40–64.80) *	58.60 (38.70–64.80) #	0.008

◊ Kruskal–Wallis test, *#^ *p* < 0.05 Mann–Whitney U test: * asthma vs. controls, # COPD vs. controls, ^ asthma vs. COPD.

## Supplementary Materials

The following are available online at https://www.mdpi.com/2073-4409/9/9/1944/s1, Figure S1. TSLP, IL-33, and IL-17A mRNA expression in moDCs after IL-13 or poly I:C stimulation in di- and triple-co-culture schemes in control subjects, patients with asthma, and patients with COPD. Figure S2. CHI3L1, IL-12p40, and TNF-α mRNA expression in moDCs after IL-13 or poly I:C stimulation in di- and triple-co-culture schemes in control subjects, patients with asthma, and patients with COPD. Figure S3. IL-1β, IL-6, and IL-8 mRNA expression in moDCs after IL-13 or poly I:C stimulation in di- and triple-co-culture schemes in control subjects, patients with asthma, and patients with COPD.

## References

[1] Kool M., Lambrecht B.N. (2007). Dendritic cells in asthma and COPD: Opportunities for drug development. Curr. Opin. Immunol..

[2] Hammad H., Lambrecht B.N. (2008). Dendritic cells and epithelial cells: Linking innate and adaptive immunity in asthma. Nat. Rev. Immunol..

[3] Whitsett J.A., Alenghat T. (2015). Respiratory epithelial cells orchestrate pulmonary innate immunity. Nat. Immunol..

[4] Parker D., Prince A. (2011). Innate immunity in the respiratory epithelium. Am. J. Respir. Cell Mol. Biol..

[5] Kool M., Willart M.A.M., Van Nimwegen M., Bergen I., Pouliot P., Virchow J.C., Rogers N., Osorio F., Reis e Sousa C., Reis E. (2011). An unexpected role for uric acid as an inducer of T helper 2 cell immunity to inhaled antigens and inflammatory mediator of allergic asthma. Immunity.

[6] Divekar R., Kita H. (2015). Recent advances in epithelium-derived cytokines (IL-33, IL-25, and thymic stromal lymphopoietin) and allergic inflammation. Curr. Opin. Allergy Clin. Immunol..

[7] Smith S.G., Gugilla A., Mukherjee M., Merim K., Irshad A., Tang W., Kinoshita T., Watson B., Oliveria J.-P., Comeau M. (2015). Thymic stromal lymphopoietin and IL-33 modulate migration of hematopoietic progenitor cells in patients with allergic asthma. J. Allergy Clin. Immunol..

[8] Walter M.J., Kajiwara N., Karanja P., Castro M., Holtzman M.J. (2001). Interleukin 12 p40 production by barrier epithelial cells during airway inflammation. J. Exp. Med..

[9] Park J.-A., Drazen J.M., Tschumperlin D.J. (2010). The chitinase-like protein YKL-40 is secreted by airway epithelial cells at base line and in response to compressive mechanical stress. J. Biol. Chem..

[10] Park S.-J., Nakagawa T., Kitamura H., Atsumi T., Kamon H., Sawa S.-I., Kamimura D., Ueda N., Iwakura Y., Ishihara K. (2004). IL-6 regulates in vivo dendritic cell differentiation through STAT3 activation. J. Immunol..

[11] Elder M.J., Webster S.J., Williams D.L., Gaston J.S.H., Goodall J.C. (2016). TSLP production by dendritic cells is modulated by IL-1β and components of the endoplasmic reticulum stress response. Eur. J. Immunol..

[12] Obregon C., Rothen-Rutishauser B., Gerber P., Gehr P., Nicod L.P. (2009). Active uptake of dendritic cell-derived exovesicles by epithelial cells induces the release of inflammatory mediators through a TNF-alpha-mediated pathway. Am. J. Pathol..

[13] Watanabe N., Hanabuchi S., Soumelis V., Yuan W., Ho S., de Waal Malefyt R., Liu Y.-J. (2004). Human thymic stromal lymphopoietin promotes dendritic cell-mediated CD4+ T cell homeostatic expansion. Nat. Immunol..

[14] Kitajima M, Lee HC, Nakayama T, Ziegler SF (2011). TSLP enhances the function of helper type 2 cells. Eur. J. Immunol..

[15] Ito T., Wang Y.-H., Duramad O., Hori T., Delespesse G.J., Watanabe N., Qin F.X.-F., Yao Z., Cao W., Liu Y.-J. (2005). TSLP-activated dendritic cells induce an inflammatory T helper type 2 cell response through OX40 ligand. J. Exp. Med..

[16] Elder M.J., Webster S.J., Fitzmaurice T.J., Shaunak A.S.D., Steinmetz M., Chee R., Mallat Z., Cohen E.S., Williams D.L., Gaston J.S.H. (2019). Dendritic Cell-Derived TSLP Negatively Regulates HIF-1α and IL-1β During Dectin-1 Signaling. Front. Immunol..

[17] Spadoni I., Iliev I.D., Rossi G., Rescigno M. (2012). Dendritic cells produce TSLP that limits the differentiation of Th17 cells, fosters Treg development, and protects against colitis. Mucosal Immunol..

[18] Besnard A.-G., Togbe D., Guillou N., Erard F., Quesniaux V., Ryffel B. (2011). IL-33-activated dendritic cells are critical for allergic airway inflammation. Eur. J. Immunol..

[19] Rank M.A., Kobayashi T., Kozaki H., Bartemes K.R., Squillace D.L., Kita H. (2009). IL-33-activated dendritic cells induce an atypical TH2-type response. J. Allergy Clin. Immunol..

[20] Su Z., Lin J., Lu F., Zhang X., Zhang L., Gandhi N.B., de Paiva C.S., Pflugfelder S.C., Li D.-Q. (2013). Potential autocrine regulation of interleukin-33/ST2 signaling of dendritic cells in allergic inflammation. Mucosal Immunol..

[21] Salvatore G., Bernoud-Hubac N., Bissay N., Debard C., Daira P., Meugnier E., Proamer F., Hanau D., Vidal H., Aricò M. (2015). Human monocyte-derived dendritic cells turn into foamy dendritic cells with IL-17A. J. Lipid Res..

[22] (2018). Global Strategy for Asthma Management and Prevention. www.ginasthma.org.

[23] (2018). Global Strategy for the Diagnosis, Management and Prevention of COPD, Global Initiative for Chronic Obstructive Lung Disease (GOLD). http://goldcopd.org/.

[24] Paplinska-Goryca M., Misiukiewicz-Stepien P., Nejman-Gryz P., Proboszcz M., Mlacki M., Gorska K., Krenke R. (2020). Epithelial-macrophage-dendritic cell interactions impact alarmins expression in asthma and COPD. Clin. Immunol..

[25] Ying S., O’Connor B., Ratoff J., Meng Q., Mallett K., Cousins D., Robinson D., Zhang G., Zhao J., Lee T.H. (2005). Thymic stromal lymphopoietin expression is increased in asthmatic airways and correlates with expression of Th2-attracting chemokines and disease severity. J. Immunol..

[26] Isaksen D.E., Baumann H., Zhou B., Nivollet S., Farr A.G., Levin S.D., Ziegler S.F. (2002). Uncoupling of proliferation and Stat5 activation in thymic stromal lymphopoietin-mediated signal transduction. J. Immunol..

[27] Soumelis V., Reche P.A., Kanzler H., Yuan W., Edward G., Homey B., Gilliet M., Ho S., Antonenko S., Lauerma A. (2002). Human epithelial cells trigger dendritic cell mediated allergic inflammation by producing TSLP. Nat. Immunol..

[28] Nakamura Y., Miyata M., Ohba T., Ando T., Hatsushika K., Suenaga F., Shimokawa N., Ohnuma Y., Katoh R., Ogawa H. (2008). Cigarette smoke extract induces thymic stromal lymphopoietin expression, leading to T(H)2-type immune responses and airway inflammation. J. Allergy Clin. Immunol..

[29] Rate A., Bosco A., McKenna K.L., Holt P.G., Upham J.W. (2012). Airway epithelial cells condition dendritic cells to express multiple immune surveillance genes. PLoS ONE.

[30] Arellano-Orden E., Calero-Acuña C., Moreno-Mata N., Gómez-Izquierdo L., Sánchez-López V., López-Ramírez C., Tobar D., López-Villalobos J.L., Gutiérrez C., Blanco-Orozco A. (2016). Cigarette Smoke Decreases the Maturation of Lung Myeloid Dendritic Cells. PLoS ONE.

[31] Vassallo R., Kroening P.R., Parambil J., Kita H. (2008). Nicotine and oxidative cigarette smoke constituents induce immune-modulatory and pro-inflammatory dendritic cell responses. Mol. Immunol..

[32] Givi M.E., Peck M.J., Boon L., Mortaz E. (2013). The role of dendritic cells in the pathogenesis of cigarette smoke-induced emphysema in mice. Eur. J. Pharmacol..

[33] Freeman C.M., Martinez F.J., Han M.K., Ames T.M., Chensue S.W., Todt J.C., Arenberg D.A., Meldrum C.A., Getty C., McCloskey L. (2009). Lung dendritic cell expression of maturation molecules increases with worsening chronic obstructive pulmonary disease. Am. J. Respir. Crit. Care Med..

[34] Anzalone G., Albano G.D., Montalbano A.M., Riccobono L., Bonanno A., Gagliardo R., Bucchieri F., Marchese R., Moscato M., Profita M. (2018). IL-17A-associated IKK-α signaling induced TSLP production in epithelial cells of COPD patients. Exp. Mol. Med..

[35] Yamada H., Hida N., Masuko H., Sakamoto T., Hizawa N. (2020). Effects of Lung Function-Related Genes and TSLP on COPD Phenotypes. COPD.

[36] Zhang K., Shan L., Rahman M.S., Unruh H., Halayko A.J., Gounni A.S. (2007). Constitutive and inducible thymic stromal lymphopoietin expression in human airway smooth muscle cells: Role in chronic obstructive pulmonary disease. Am. J. Physiol. Lung Cell. Mol. Physiol..

[37] Eiwegger T., Akdis C.A. (2011). IL-33 links tissue cells, dendritic cells and Th2 cell development in a mouse model of asthma. Eur. J. Immunol..

[38] Turnquist H.R., Thomson A.W. (2009). IL-33 broadens its repertoire to affect DC. Eur. J. Immunol..

[39] Tjota M.Y., Hrusch C.L., Blaine K.M., Williams J.W., Barrett N.A., Sperling A.I. (2014). Signaling through FcRγ-associated receptors on dendritic cells drives IL-33-dependent TH2-type responses. J. Allergy Clin. Immunol..

[40] Schön M.P., Erpenbeck L. (2018). The Interleukin-23/Interleukin-17 Axis Links Adaptive and Innate Immunity in Psoriasis. Front. Immunol..

[41] Lin A.M., Rubin C.J., Khandpur R., Wang J.Y., Riblett M., Yalavarthi S., Villanueva E.C., Shah P., Kaplan M.J., Bruce A.T. (2011). Mast cells and neutrophils release IL-17 through extracellular trap formation in psoriasis. J. Immunol..

[42] Ishigame H., Kakuta S., Nagai T., Kadoki M., Nambu A., Komiyama Y., Fujikado N., Tanahashi Y., Akitsu A., Kotaki H. (2009). Differential roles of interleukin-17A and -17F in host defense against mucoepithelial bacterial infection and allergic responses. Immunity.

[43] Ouyang W., Kolls J.K., Zheng Y. (2008). The biological functions of T helper 17 cell effector cytokines in inflammation. Immunity.

[44] Blank F., Von Garnier C., Obregon C., Rothen-Rutishauser B., Gehr P., Nicod L. (2008). Role of dendritic cells in the lung: In vitro models, animal models and human studies. Expert Rev. Respir. Med..

[45] Blom R.A.M., Erni S.T., Krempaská K., Schaerer O., Van Dijk R.M., Amacker M., Moser C., Hall S.R.R., Von Garnier C., Blank F. (2016). A Triple Co-Culture Model of the Human Respiratory Tract to Study Immune-Modulatory Effects of Liposomes and Virosomes. PLoS ONE.

