# Supplementary material for: The Expressions of TSLP, IL-33, and IL-17A in Monocyte Derived Dendritic Cells from Asthma and COPD Patients are Related to Epithelial–Macrophage Interactions
Source: Cells. 2020 Aug 22;9(9):1944. doi: 10.3390/cells9091944 (PMC7565129; doi:10.3390/cells9091944)
Supplement: Supplementary file 1 [file cells-09-01944-s001.pdf]

moDCs TSLP, IL-33 and IL-17A mRNA expression in multi co-cultures with or without IL-13 or poly I:C stimulation in control group.

TSLP, IL-33 and IL-17A mRNA expression in moDCs after IL-13 or poly I:C stimulation in di- and triple co-culture schemes in control subjects is presented in Figure S1.

Stimulation with IL-13 did not change TSLP mRNA expression in any of the cultures. An increased TSLP mRNA expression was observed after poly I:C stimulation in moDCs from triple co-culture (3.56 fold change (2.50-5.37 fold change)) compared to moDCs alone.

The highest (insignificant) IL-33 mRNA expression was noted in moDCs/epithelium+poly I:C group (274.25 fold change (0.14-1306.34 fold change)) compared to moDCs alone (0.40 fold change (0.06-1.65 fold change)) and moDCs co-cultivated with epithelium (8.17 fold change (0.52-3887.15 fold change)).

Stimulation with IL-13 insignificantly increased IL17A mRNA expression in moDCs co-cultivated with epithelium (3.22 fold change (1.36-6.50 fold change)) as well as epithelium+moMφs (5.74 fold change (1.96-8.78 fold change)). This was not observed in poly I:C stimulated cells.

CHI3L1, IL-12p40, TNF-α IL-1β, IL-6 and IL-8 expressions in moDCs after IL-13 or poly I:C stimulation in multi co-culture schemes in control subjects are shown in Figure S2 and S3.

moDCs TSLP, IL-33 and IL-17A mRNA expression in multi co-cultures with or without IL-13 or poly I:C stimulation in asthma group.

TSLP, IL-33 and IL-17A mRNA expression in moDCs after IL-13 or poly I:C stimulation in di and triple co-culture schemes in asthma patients is presented in Figure S1.

IL-13 stimulation tended to decrease TSLP expression mRNA, however, the change was not significant in any of co-cultivation schemes. The increased TSLP mRNA expression was observed after poly I:C stimulation in moDCs from triple co-cultures (3.68 fold change (1.12-15.72 fold change) compared to moDCs co-cultivated with epithelial cells.

IL-13 or poly I:C stimulation did not influence IL-33 mRNA expression in any of the investigated models.

CHI3L1, IL-12p40, TNF-α IL-1β, IL-6 and IL-8 expressions in moDCs after IL-13 or poly I:C stimulation in multi co-culture schemes in asthma patients are shown in Figure S2 and S3.

TSLP, IL-33 and IL-17A mRNA expression in moDCs in multi co-culture schemes with or without IL-13 or poly I:C stimulation in COPD group.

TSLP, IL-33 and IL-17A mRNA expression in moDCs after IL-13 or poly I:C stimulation in di and triple co-culture schemes in COPD patients is presented in Figure S1.

There were no differences in TSLP, IL-33 or IL-17A mRNA expression in COPD group regardless of co-culture type or stimulation used.

CHI3L1, IL-12p40, TNF-α IL-1β, IL-6 and IL-8 mRNA expressions in moDCs after IL-13 or poly I:C stimulation in multi co-culture schemes in COPD patients are shown in Figure S2 and S3.

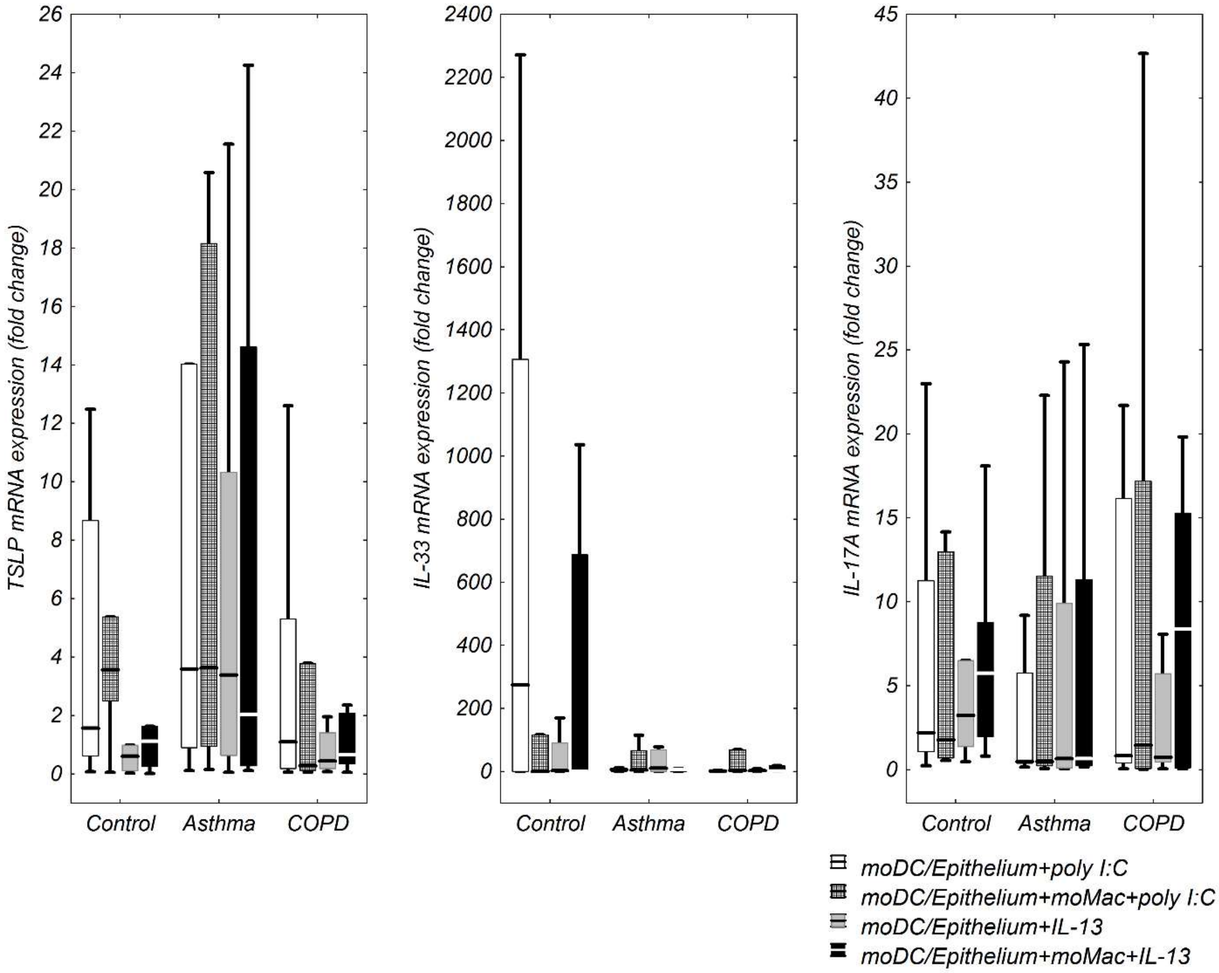

Figure S1. TSLP, IL-33 and IL-17A mRNA expression in moDCs after IL-13 or poly I:C stimulation in di- and trile-co-culture schemes in control subjects, asthma and COPD patients. The data are shown as non-outlier range (whiskers), interquartile range (box) and median (line).

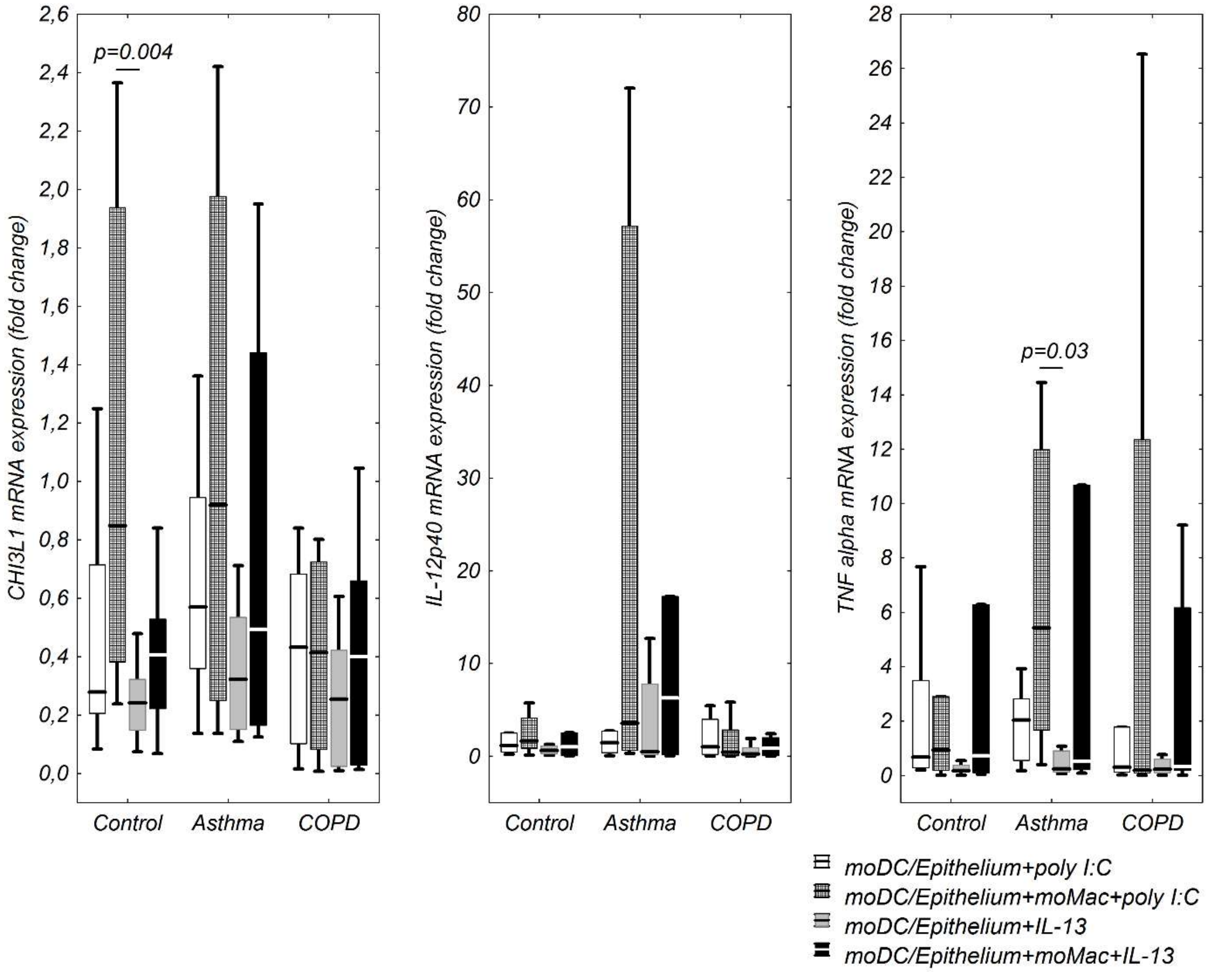

Figure S2. CHI3L1, IL-12p40 and TNF-α mRNA expression in moDCs after IL-13 or poly I:C stimulation in di- and trile-co-culture schemes in control subjects, asthma and COPD patients. The data are shown as non-outlier range (whiskers), interquartile range (box) and median (line).

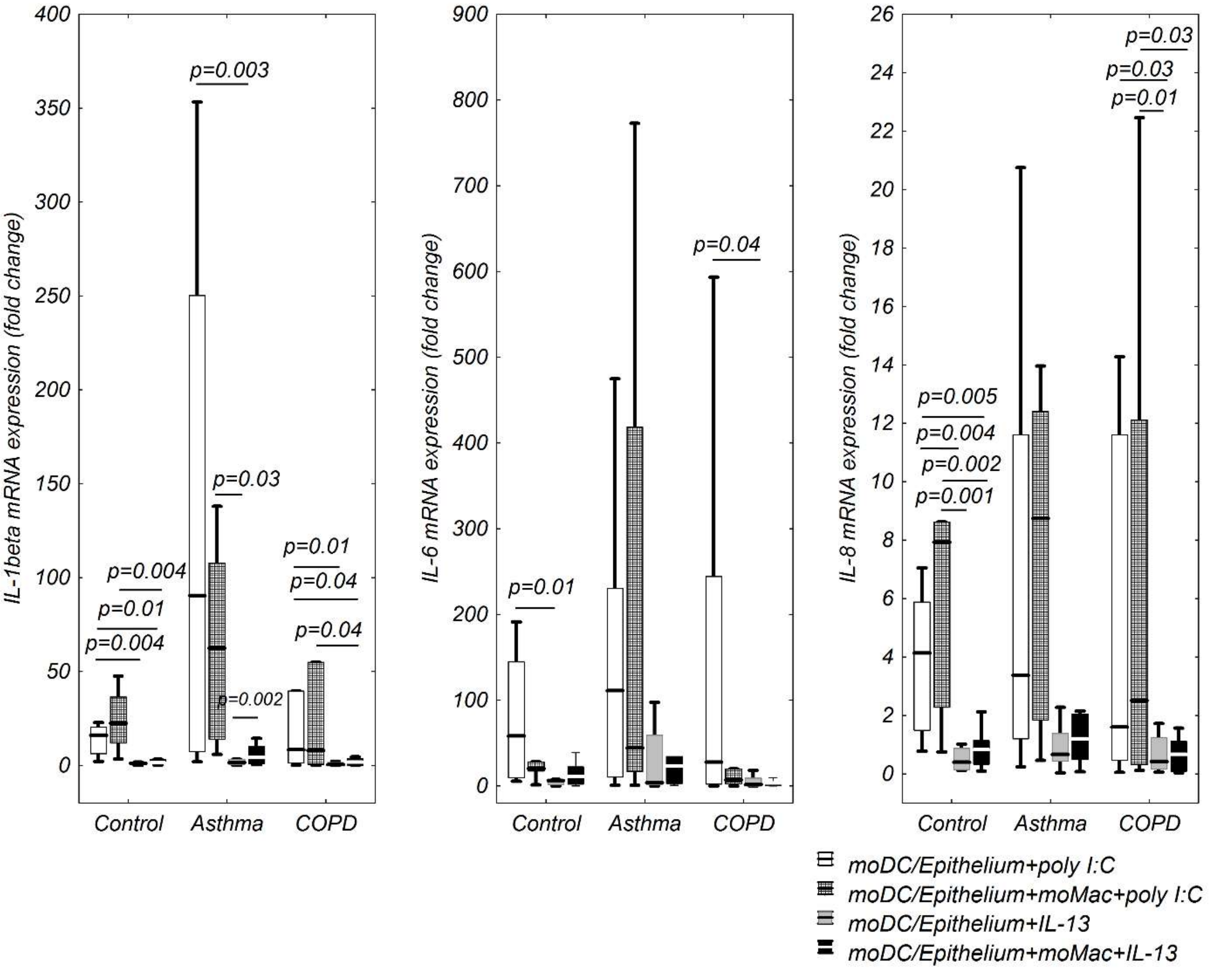

Figure S3. IL-1β, IL-6 and IL-8 mRNA expression in moDCs after IL-13 or poly I:C stimulation in di- and trile-co-culture schemes in control subjects, asthma and COPD patients. The data are shown as non-outlier range (whiskers), interquartile range (box) and median (line).
